# Job crafting interventions: what works, for whom, why, and in which contexts? Research protocol for a systematic review with coincidence analysis

**DOI:** 10.1186/s13643-023-02170-z

**Published:** 2023-01-20

**Authors:** Marta Roczniewska, Anna Rogala, Magdalena Marszałek, Henna Hasson, Arnold B. Bakker, Ulrica von Thiele Schwarz

**Affiliations:** 1 PROCOME Research Group, Medical Management Centre, Department of Learning, Informatics, Management and Ethics, Karolinska Institutet, Stockholm, Sweden; 2 Faculty of Psychology in Sopot, SWPS University of Social Sciences and Humanities, Sopot, Poland; 3 Faculty of Psychology in Warsaw, SWPS University of Social Sciences and Humanities, Warsaw, Poland; 4 Centre for Epidemiology and Community Medicine, Stockholm, Sweden; 5 Center of Excellence for Positive Organizational Psychology, Erasmus University Rotterdam, Rotterdam, The Netherlands; 6 Department of Industrial Psychology and People Management, University of Johannesburg, Johannesburg, South Africa; 7 School of Health, Care and Social Welfare, Mälardalen University, Västerås, Sweden

## Abstract

**Background:**

Recent challenges in the working world that resulted from the pandemic and technological advances have underlined the importance of flexibility in how jobs are designed. Job crafting (JC) refers to self-initiated changes that employees introduce to their jobs to optimize their job design and increase the fit between the job and their needs and preferences. These behaviors can be stimulated by job crafting training interventions, which aim to change how individual employees design, organize, or manage their work. However, since the interventions are implemented in various ways, we do not know which context and intervention factors are necessary or sufficient to achieve desired outcomes. Without this knowledge, benefitting from the potential of job crafting interventions is limited. The overall aim of this project will be to investigate what combinations of context, intervention, and mechanism factors are linked with effective JC interventions. Specifically, we will detect what factors are minimally sufficient and/or necessary to produce a successful JC intervention, how they combine, as well as what are the multiple alternative paths to their success.

**Methods:**

We will perform a systematic review of the JC interventions literature combined with coincidence analysis (CNA). We will search electronic databases of journals and utilize Rayyan software to make decisions regarding inclusion. Data regarding context (e.g., fit), intervention (e.g., types of activities), mechanisms (e.g., intention implementation), and outcomes (e.g., employee well-being, job performance) will be extracted using a pre-piloted form and coded into a crisp-set (factor present vs. absent). Analyses will be carried out using the CNA package in R.

**Discussion:**

This review will address gaps in knowledge about the context, intervention, and mechanism-related factors that may impact the effects of JC interventions. Consequently, this review will help develop a program theory for JC interventions that explains what works, how and under which circumstances. Applying CNA to synthesize these complex solutions across multiple studies provides an innovative method that may be used in future review attempts evaluating the implementation of interventions. Finally, our synthesis will provide knowledge relevant to organizational practitioners and scholars who want to implement JC interventions.

**Trial registration:**

https://osf.io/2g6yx

**Supplementary Information:**

The online version contains supplementary material available at 10.1186/s13643-023-02170-z.

## Background

Economic pressures, technological advances, and changes within organizations themselves emphasize the importance of flexibility in the ways that jobs are performed in modern workplaces. To address these challenges, organizations often employ a top-down job redesign approach in which management optimizes the demands and resources of employees’ jobs to obtain desired organizational outcomes [1]. However, such top-down strategies are unsuitable to keep up with the rapid pace of current changes and often fail to recognize the diversity of the workforce and growing job specialization among employees. A top-down redesign may result in a poor fit between employees’ needs or abilities and the organizational environment, which poses a risk of low job satisfaction, increased turnover, and work-related ill-health among employees [2]. These negative outcomes jeopardize sustainable employment (i.e., the extent to which workers are able and willing to remain working now and in the future [3]), which is one of the main challenges in Europe [4]. Without proper approaches, organizations’ business goals are at risk.

Overall, societal changes, dynamic workplaces, and a diverse workforce, combined with the inadequacy of traditional top-down approaches, call for solutions that are driven by employees themselves. Employee-driven job redesign enables employees to “take charge” of their own work to achieve better person-job fit. Proactive employee job redesign is called *job crafting* (JC). This bottom-up strategy refers to self-initiated changes that employees introduce to their jobs to allow them to deal more effectively with the demands of the changing work environment [5]. With JC, employees proactively shape their jobs and align them with their own needs [6, 7]. There are two main approaches to how JC is framed. Wrzesniewski and Dutton [6] describe JC as proactive changes in physical, relational, and cognitive job aspects. Simply put, people who craft their jobs change their task boundaries, social interactions (e.g., with colleagues or clients), and also how they think about their role. The second approach frames JC within the Job Demands-Resources (JD-R) model [8–10] and defined it as behaviors aimed at changing two types of job characteristics: job resources and job demands to find a better person-job fit [7]. Specifically, employees may seek more resources, increase challenging job demands, and reduce hindering job demands [11].

JC emerges as a possible solution to the challenges of the current job redesign. First, employees can flexibly react to organizational changes affecting their jobs more quickly as compared to top-down actions. Second, knowing the core of their jobs enables employees to shape them more effectively. Finally, the redesign accounts for individual needs and preferences and thus, is instrumental to achieving person-job fit. Hence, both employees and organizations could profit from employee proactivity in job redesign. Research on the outcomes of JC confirms its beneficial effects. JC has been linked with higher employee job satisfaction [12], engagement [13], job performance [14], person-job fit [15], work meaning [16], as well as lower job burnout [12] and intentions to leave [13]. Employees who craft their jobs adapt to changes better [17, 18]. Such boosted adaptability was observed in the recent COVID-19 pandemic: when employees had to redesign their jobs to match the new circumstances, deal with the new demands, and achieve work-life balance, those who engaged in job crafting, dealt with telework better [19]. Overall, JC may be linked with more sustainable employment[5, 20].

Given the positive outcomes of JC, it is important to understand how organizations can stimulate such proactivity. While JC concerns employees’ self-initiated actions, it can be supported through actions undertaken by an organization. Thus, organizations can introduce *job crafting training interventions*, by means of which employees learn about job crafting and are stimulated to use it in their work [21–23]. The research on job crafting interventions is relatively new. The Job Crafting Exercise booklet was first described by Wrzesniewski and colleagues in 2013 [22]. Another wave of job crafting, which derived from the JD-R model, proposed a corresponding job crafting training workshop in 2015[21]. To the best of our knowledge, there are two existing literature reviews published in 2019 that focus specifically on examining the effects of job crafting interventions. First, a systematic review summarized eight empirical quantitative studies examining the effects of job crafting interventions and demonstrated mixed findings for different types of job crafting behaviors, well-being variables, and job performance [24]. Second, a meta-analysis of 14 JC interventions revealed overall statistically significant results on global JC (i.e., total JC score composed of all dimensions), but not for all job crafting types when investigated separately; it also demonstrated an effect of the interventions on work engagement and on contextual performance [25].

While these reviews have been valuable, they focused on answering *whether* job crafting interventions worked (i.e., led to an increase in JC behaviors post intervention), but did not set out to answer the important questions of *why* they worked, *when,* and *for whom*. Yet, these process and context factors should be part of the evaluation to better explain the success and failure of workplace interventions [26]. Little is known about which employees may benefit from JC interventions and how these interventions are implemented at workplaces. In addition, more knowledge is needed on what workplace characteristics might impact the implementation and effect of such interventions. An exception was the moderator analyses in the aforementioned meta-analysis, which looked at two factors: the occupational sector and an intervention objective (individual goal vs. individual and organizational goals combined) [25]. Yet, these are only two possible aspects (related to context and intervention activities, respectively) that may affect the success of the JC intervention. Research has established an extensive list of potential contextual and intervention-related factors that may affect implementation and the intervention outcomes [27]. To move the field forward, we need to better understand what combinations of factors are relevant to elicit successful implementation of job crafting in the workplace as an outcome of the intervention. Job crafting interventions published in the literature are heterogeneous: Depending on the underlying JC theory, the contents of these interventions differ, as do the methods used (e.g., workshops, feedback sessions, booklets, medium used). These differences make it difficult to determine the true potential of a JC intervention by testing an overall meta-analytical effect. In addition, the heterogeneous context does not need to be viewed as a confounding influence that should be controlled, but rather as a factor that influences how the intervention brings about its outcome through certain mechanisms [28]. Additionally, JC interventions may have unique implementation characteristics. Namely, JC interventions combine a mix of approaches: by means of decisions and actions initiated by management that take *a top-down* form, they aim to introduce a continuous *bottom-up* change among individuals. Given that JC behaviors differ between individuals and may go unnoticed by managers[6], identifying elements of effective support for JC interventions is a challenge. Although extensive literature exists on the implementation of organizational interventions [29], less is known about introducing such unique combinations [30].

## Aim and research questions

Overall, knowledge on JC interventions and their effects is still scarce and spread across distinct approaches. We have yet to determine which context and intervention factors are necessary or sufficient to achieve desired outcomes. The general aim of this project will be to investigate what combinations of context, intervention, and mechanism factors are linked with effective JC interventions. Specifically, we will detect what factors are minimally sufficient and/or necessary to produce a successful JC intervention, as well as the multiple alternative paths to their success. The following research questions (RQ) will be investigated:

- RQ1. What program theories have been used for JC interventions?
- RQ2. What activities have been used in JC interventions?
- RQ3. What have been the proximal and distal outcomes of JC interventions for performance and health and well-being?
- RQ4. Which Context-Intervention-Mechanism (CIM) factors are sufficient for successful JC interventions?
- RQ5. Which Context-Intervention-Mechanism (CIM) factors are necessary for successful JC interventions?
- RQ6. How do the factors combine to produce successful JC interventions?
- RQ7. What are the (multiple) paths to successful JC interventions?
- RQ8. Is a successful JC intervention (i.e., an increase in JC) a sufficient and necessary condition for effects for well-being and performance?

## Methods

This protocol is reported according to the Preferred Reporting Items for Systematic Reviews and Meta-analysis Protocols (PRISMA-P) 2015 checklist (see Additional file 1) [31].

### Approach

We will perform a systematic review of JC literature and, to be able to address our research questions, we will apply CIMO logic, which aims to determine the combinations showing that in context C, an intervention I invokes generative mechanisms M that produces outcome O. In this way, we can establish what works, how, in what context and for whom. This will be achieved by applying a mathematical, cross-case method called Coincidence Analysis (CNA), which belongs to a broader class of Configurational Comparative Methods (CCMs; [32]). These methods have been designed explicitly to support causal inferences, answer research questions about combinations of conditions that are minimally necessary or sufficient for an outcome, and to identify the possible presence of multiple causal paths to an outcome [33]. CNA can be applied to large-n as well as small-n sets. It has recently been used in multiple projects which aimed at discovering minimally sufficient and necessary factors affecting successful implementation, as well as their combinations and multiple paths to outcomes [32, 34, 35]. Moreover, CCMs were applied in a recent Cochrane Review which aimed at identifying conditions associated with a successful implementation of school-based interventions for asthma self-management [36]. Finally, configurational methods have been included as a relevant method for implementation research in the Handbook on Implementation Science [37, 38].

CNA operates based on the Boolean properties of causation, which encompass three dimensions of complexity. The first is *conjunctivity*: to bring about an outcome, several conditions must be jointly present. For example, the analysis may demonstrate that a job crafting intervention is successful when individuals are white-collar employees AND are provided with tasks to perform (homework) between workshop sessions AND when they are reminded to engage in these tasks. The second dimension of complexity is *disjunctivity (equifinality)*, which means that different paths can lead to the same outcome. For instance, a success in job crafting interventions can be achieved when there is alignment of the intervention with organizational aims and when reminders of JC activities are sent OR when there is no alignment present, but managers have been trained alongside employees. The third dimension of complexity is *sequentiality*, which points to a possibility that outcomes tend to produce further outcomes, propagating causal influence along causal chains. For instance, an increase in job crafting as a result of the intervention can, in turn, lead to an increase in job performance. Thus, CNA analysis will be instrumental in answering our questions about sufficient (RQ4) and necessary (RQ5) factors, as well as their combinations (RQ6), and potential multiple paths (RQ7) or sequences (RQ8) that lead to the intervention success.

### Eligibility criteria

To systematize study selection, we will use the PICO (Population; Intervention;

Comparison; Outcome) approach. Specifically, to be included in our review, the study:

- Has to involve active employees (i.e., not a student sample, not persons on leave) as participants (P);
- Has to be a job crafting intervention (I);
- Contains a comparator (intragroup, between group, or control group) (C); and
- Contains measures of job crafting (general or specific types) as a proximal intervention outcome and/or contains health and well-being or performance as distal outcomes (O).

### Search terms and strategy

The search will be executed by a professional team from the Karolinska Institutet (KI) library that specializes in literature reviews. It will be conducted in the following databases: PsycINFO, Web of Science, Academic Search Complete, MEDLINE, and CINAHL. The search will be conducted for research published with a lower time limit of 2001 because the first article about job crafting was published in 2001 (see draft search strategy in Additional file 2). The search terms will be based on the topic of this review, i.e., job crafting (e.g., “job crafting” as well as its specific types, such as “seeking challenges” or “increasing structural job resources”) and the intervention (e.g., “intervention,” “experiment,” or “workshop”).^1^ Additionally, we will inspect the references of two published literature reviews on job crafting interventions: a systematic review [24] and a meta-analysis with utility analysis [25].

### Review and extraction

All retrieved references will be uploaded to open-source Rayyan software [39]. Abstracts of the retrieved studies will be screened independently by 2 team members to identify studies that meet the inclusion criteria. Then, full texts of these studies will be independently assessed by the two authors for eligibility. Discrepancies in the screening at these two stages will be resolved by discussion, and when needed, a third person will be consulted.

A standardized, pre-piloted form will be used to extract data from the included studies. We conducted a feasibility test for 5 published articles that contained a job crafting intervention to derive factors of relevance within the CIMO logic. Four groups of data will be extracted: Context (e.g., alignment, participants, co-occurring changes), Intervention (e.g., workshop duration, action plans, feedback), Mechanism (e.g., theory of planned behavior, job demands-resources model, experiential learning), and Outcomes (e.g., effects for job crafting, health and well-being, performance). Each record will be extracted by two independent extractors in duplicate to ensure consistency and a high quality. Additional factors will be inductively added during data retrieval if they appear relevant for the CIMO logic. Authors will be contacted via e-mail to clarify or provide more information on the conducted intervention.

### Risk of bias

Following the meta-analysis of JC interventions [25], we will use the Cochrane Collaboration tool [40] to evaluate the risk of bias. We will consider selection bias (i.e., sequence generation, allocation concealment), performance bias (blinding of participants/personnel), detection bias (blinding of outcome assessment), attrition bias (i.e., incomplete outcome data), reporting bias (selective outcome reporting), and other potential sources of bias. Each domain will be evaluated for each intervention as either a low, high, or unclear risk of bias. Consequently, the more domains are assigned low risk in a particular study, the higher their quality.

### Synthesis/data analysis

Regularity theoretic causation, which will be applied in this synthesis using CNA, is a relation that holds between factors taking on specific *values* [33]. In our synthesis, we will use a crisp set, where each case will take on 0 and 1 as possible values for each factor. Thus, the extracted information for each factor will be coded as 1 (reflecting a presence of a factor) and 0 (reflecting a factor absence) based on clearly defined criteria.

The definitions and criteria will be developed by the research team, pilot-tested based on a sample of relevant articles, and refined if needed. Again, coding will be done by two independent coders in duplicate and codes will be compared. Table 1 presents examples of factors for each CIM category as well as coding criteria.

**Table 1:** Examples of potential factors in the categories of Context, Intervention, and Mechanism with coding criteria

*Category and factor*	**Coding criteria: Assign 1 when**	**Coding criteria: Assign 0 when**
**Context (C)**		
** Fit**	The intervention is described as implemented in response to a specific organizational need	The intervention is implemented without consideration of a specific need
** Presence of co-occurring changes**	Changes co-occurring with the intervention are mentioned	No co-occurring changes are described
** Homogeneity of the profession**	Intervention participants all have the same/similar job (e.g., all are nurses, all are teachers, etc.)	Participants in the interventions have mixed jobs/professions
**Intervention (I)**		
** Job analysis**	The intervention included an analysis of resources (mental, physical, social, and organizational factors, enabling professional goals to be achieved and reduction of costs), demands (mental, physical, social, and organizational, requiring effort or skills from the employee), organizational barriers, and/or constraints	The intervention activities did not include any job analysis pertaining to job demands, resources, organizational barriers, or constraints
** Action plans**	The intervention involved planning future JC activities by participants	No plans for JC activities were created by participants as a result of an intervention
** Reminders**	The participants received reminders about fulfilling action plans or post-workshop homework	No reminders were provided for participants about fulfilling action plans or post-workshop homework
**Mechanism (M)**		
Job demands-resources model	The authors indicate that their intervention study and hypotheses are based on the JD-R model	No or theories other than the JD-R model are mentioned
Self-determination theory	The authors clearly indicate that their intervention study and hypotheses are based on self-determination theory	No or theories other than self-determination theory are mentioned
Theory of planned behavior	The authors clearly indicate that their intervention study and hypotheses are based on the theory of planned behavior	No, or other, theories than the theory of planned behavior are mentioned

Data analysis will be performed in a devoted CNA package in R. The output will be interpreted in terms of conditions sufficient (RQ4), conditions necessary (RQ5), their combinations (RQ6), and possible alternative paths to the outcome (RQ7). We will also investigate whether a success in a JC intervention is sufficient and/or necessary with an increase in well-being and performance after the intervention (RQ8). The interpretation of the output from configurational methods will be done with specific attention to consistency (degree to which the solution always yields the expected outcome; [33]) and coverage (degree to which the solution covers all cases; [33]) of the results, non-redundancy of the factors, and consistency with logic, theory, and prior knowledge.

The strength of the body of evidence will be assessed using relevant domains from the Guide to Community Preventive Services [41], which serves as a framework to evaluate “confidence that changes in outcomes are attributable to the interventions” (p. 38). This framework is suitable for narrative synthesis. Based on this framework, evidence will be rated as strong, sufficient, or insufficient.

## Discussion

With JC repeatedly being linked with positive employee and organizational outcomes [13] it is not surprising that JC has drawn a lot of attention from scholars and practitioners alike. By considering individuals as active creators of their jobs, JC interventions may be a promising strategy for securing organizational and employee sustainability. However, to fully capitalize on the benefits of JC, organizations need to understand how to stimulate JC in their environments. Unfortunately, knowledge about JC interventions is still scarce and spread across distinct theoretical perspectives. It is not yet clear when and how these interventions produce desired outcomes. Without this knowledge, it may be futile to introduce JC interventions into everyday organizational practice. Thus, it is vital to understand when, how and for whom these interventions lead to desired outcomes. With this review, we will provide knowledge regarding JC interventions that have a chance to move the area forward. The results will illuminate which combinations of factors are necessary for succeeding with implementation of JC interventions, thereby providing organizations with guidelines for achieving sustainable effects of JC interventions. Additionally, given the uniqueness of JC interventions in that they are organizational interventions for individuals to intervene on their jobs themselves, this synthesis will allow us to better understand the uniqueness of such solutions and their implementation characteristics. The synthesis will also allow us to investigate what sort of information about the CIM factors in the JC interventions is volunteered by the researchers in the first place and may lead to proposing guidelines on how to report them more transparently by including information about all relevant factors.

By complementing the systematic review with CNA, we develop a novel way to assess how CIMO configurations play out to produce an aspired outcome. Thereby, this project will make a contribution both to the knowledge synthesis and the evaluation literature, as the approach can be used both for secondary data, in a synthesis, and primary data, as in an evaluation. Given the complex, multifaceted nature of organizational interventions, this approach is particularly suited to the synthesis of evidence about complexity in the implementation of JC interventions. By complementing the systematic review with CNA, the findings will be based on quantitative analyses—specifically, a Boolean algebra technique. This approach enables identification of multiple configurations that are sufficient to produce an outcome with enough consistency to illustrate that the same pathway will continue to produce the outcome. Interventions usually contain multiple components and are influenced by multiple contextual factors working through several possible mediators to achieve a sequence of outcomes. Thus, the number of possible combinations to analyze and make sense of them quickly exceeds what is possible for humans to overview. Applying a mathematical method that addresses this and additional dimensions of complexity, thus, extends the qualitative approaches often used in systematic reviews to help uncover “what works for whom and how”.

## Supplementary Information

**Additional file 1.** PRISMA-P checklist.

**Additional file 2.** Documentation for search strategies.

## Data Availability

Data sharing is not applicable to this article as this is a study protocol.

## Abbreviations

CIM: Context-Intervention-Mechanism
CCMs: Configurational Comparative Methods
CNA: Coincidence analysis
JC: Job crafting

## Footnotes

1 The final refined list of search terms will be included in the systematic review upon publishing the results.

## References

[1] Oldham GR, Hackman JR (2010). Not what it was and not what it will be: The future of job design research. J Organ Behav.

[2] Kristof-Brown AL, Zimmerman RD, Johnson EC (2005). Consequences of individuals’ fit at work: A meta-analysis of person-job, person-organization, person-group, and person-supervisor fit. Pers Psychol.

[3] van der Klink JJL, Bültmann U, Burdorf A, Schaufeli WB, Zijlstra FRH, Abma FI, et al. Sustainable employability – definition, conceptualization, and implications: A perspective based on the capability approach. Scand J Work Environ Health. 2016;42:71–9. Available from: http://www.sjweh.fi/show_abstract.php?abstract_id=3531 10.5271/sjweh.3531 26595874

[4] van den Heuvel G, Verbeek, Nold, Euler, Fishta. 371 Priority setting for future occupational safety and health (OSH) research in Europe. Occup Environ Med. 2013;70:A126.3–A127.

[5] le Blanc PM, Demerouti E, Bakker AB. How Can I Shape My Job to Suit Me Better? Job Crafting for Sustainable Employees and Organizations. In: Chmiel N, Fraccaroli F, Sverke M, editors. An Introduction to Work and Organizational Psychology [Internet]. Oxford, UK: Wiley; 2017. p. 48–63. Available from: http://doi.wiley.com/ 10.1002/9781119168058.ch3

[6] Wrzesniewski A, Dutton JE (2001). Crafting a job: Revisioning employees as active crafters of their work. Acad Manag Rev.

[7] Tims M, Bakker AB. Job crafting: Towards a new model of individual job redesign. South African J Industrial Psychol. 2010;36:1–9. Available from: http://sajip.co.za/index.php/sajip/article/view/841

[8] Demerouti E, Bakker AB, Nachreiner F, Schaufeli WB (2001). The job demands-resources model of burnout. J Appl Psychol.

[9] Bakker AB, Demerouti E. The Job Demands-Resources model: State of the art. J Manag Psychol. 2007;22:309–28. Available from: https://www.emeraldinsight.com/doi/ 10.1108/02683940710733115

[10] Bakker AB, Demerouti E. Job demands–resources theory: Taking stock and looking forward. J Occup Health Psychol [Internet]. 2017;22:273–85. Available from: http://doi.apa.org/getdoi.cfm? 10.1037/ocp0000056 10.1037/ocp0000056 27732008

[11] Tims M, Bakker AB, Derks D (2012). Development and validation of the job crafting scale. J Vocat Behav.

[12] Tims M, Bakker AB, Derks D (2013). The impact of job crafting on job demands, job resources, and well-being. J Occup Health Psychol.

[13] Rudolph CW, Katz IM, Lavigne KN, Zacher H. Job crafting: A meta-analysis of relationships with individual differences, job characteristics, and work outcomes. J Vocat Behav [Internet]. 2017;102:112–38. Available from: https://linkinghub.elsevier.com/retrieve/pii/S0001879117300477

[14] Tims M, Bakker AB, Derks D (2015). Job crafting and job performance: A longitudinal study. Eur J Work Organ Psy.

[15] Tims M, Derks D, Bakker AB (2016). Job crafting and its relationships with person-job fit and meaningfulness: A three-wave study. J Vocat Behav.

[16] Berg JM, Dutton JE, Wrzesniewski A. Job crafting and meaningful work. Purpose and meaning in the workplace. 2013. p. 81–104.

[17] Petrou P, Demerouti E, Schaufeli WB. Crafting the change: The role of employee job crafting behaviors for successful organizational change. J Manage [Internet]. 2016;18:1–27. Available from: http://jom.sagepub.com/cgi/doi/ 10.1177/0149206315624961

[18] Petrou P, Demerouti E, Schaufeli WB (2015). Job crafting in changing organizations: Antecedents and implications for exhaustion and performance. J Occup Health Psychol.

[19] Stempel CR, Siestrup K. Suddenly Telework: Job Crafting as a Way to Promote Employee Well-Being? Front Psychol. 2022;12:6561. 10.3389/fpsyg.2021.790862 PMC8795870 35095676

[20] Irfan SM, Qadeer F, Abdullah MI, Sarfraz M. Employer’s investments in job crafting to promote knowledge worker’s sustainable employability: a moderated mediation model. Personnel Rev. 2022;12:144–64. Available from: https://papers.ssrn.com/sol3/papers.cfm?abstract_id=3770645.

[21] van den Heuvel M, Demerouti E, Peeters MCW. The job crafting intervention: Effects on job resources, self-efficacy, and affective well-being. J Occup Organ Psychol. 88:511–32. Available from: http://doi.wiley.com 10.1111/joop.12128

[22] Berg J, Dutton J, Wrzesniewski A, Baker W. Job Crafting Exercise. Job Crafting Exercise. 2013.

[23] van Wingerden J, Bakker AB, Derks D. Fostering employee well-being via a job crafting intervention. J Vocat Behav. Academic Press Inc.; 2017;100:164–74.

[24] de Devotto RP, Wechsler SM. Job crafting interventions: Systematic review. Trends Psychol. 2019;27:371–83. Springer Science and Business Media Deutschland GmbH. Available from: http://www.scielo.br/scielo.php?script=sci_arttext&pid=S2358-18832019000200371&lng=en&nrm=iso&tlng=en.

[25] Oprea BT, Barzin L, Vîrgă D, Iliescu D, Rusu A. Effectiveness of job crafting interventions: a meta-analysis and utility analysis. Eur J Work Organ Psychol. 2019;28:723–41. Routledge. Available from: https://www.tandfonline.com/doi/full/10.1080/1359432X.2019.1646728.

[26] von Thiele Schwarz U, Nielsen K, Edwards K, Hasson H, Ipsen C, Savage C, et al. How to design, implement and evaluate organizational interventions for maximum impact: the Sigtuna Principles. Eur J Work Organ Psychol. 2021;30:415–27. Routledge. Available from: https://www.tandfonline.com/action/journalInformation?journalCode=pewo20. 10.1080/1359432X.2020.1803960 PMC8432268 34518756

[27] Nielsen K, Randall R. Opening the black box: Presenting a model for evaluating organizational-level interventions. European Journal of Work and Organizational Psychology [Internet]. 2013;22:601–17. Available from: http://www.tandfonline.com/doi/abs/ 10.1080/1359432X.2012.690556

[28] The Science of Evaluation: A Realist Manifesto - Ray Pawson - Google Books. Available from: https://books.google.se/books?hl=en&lr=&id=4lPgb6gtPTIC&oi=fnd&pg=PP2&dq=(Pawson,+2013&ots=vDg7GVsf83&sig=boobWIlmDxmo6KgveXmWn06nBuQ&redir_esc=y#v=onepage&q=(Pawson%2C%202013&f=false.

[29] Biron C, Karanika-Murray M, Cooper CL (2012). Improving Organizational Interventions for Stress and Well-Being.

[30] Roczniewska M, Hedberg Rundgren E, Hasson H, Bakker AB, von Thiele Schwarz U. How should job crafting interventions be implemented to make their effects last? Protocol for a group concept mapping study. Int J Environ Res Public Health. 2022;19:13922. MDPI AG. Available from: https://www.mdpi.com/1660-4601/19/21/13922/htm. 10.3390/ijerph192113922 PMC9654425 36360800

[31] Moher D, Liberati A, Tetzlaff J, Altman DG. Preferred Reporting Items for Systematic Reviews and Meta-Analyses: The PRISMA Statement. Ann Intern Med. 2009;151:264. 10.7326/0003-4819-151-4-200908180-00135 19622511

[32] Whitaker RG, Sperber N, Baumgartner M, Thiem A, Cragun D, Damschroder L, et al. Coincidence analysis: a new method for causal inference in implementation science. Implementation Sci. 2020;15:1–10. BioMed Central Ltd. Available from: https://link.springer.com/articles/10.1186/s13012-020-01070-3. 10.1186/s13012-020-01070-3 PMC7730775 33308250

[33] Baumgartner M, Thiem A (2015). Identifying Complex Causal Dependencies in Configurational Data with Coincidence Analysis. R J.

[34] Yakovchenko V, Miech EJ, Chinman MJ, Chartier M, Gonzalez R, Kirchner JAE, et al. Strategy configurations directly linked to higher hepatitis C virus treatment starts: an applied use of configurational comparative methods. Med Care. 2020;58:E31–8. Lippincott Williams and Wilkins. Available from: /pmc/articles/PMC7161717/. 10.1097/MLR.0000000000001319 PMC7161717 32187105

[35] Petrik AF, Green B, Schneider J, Miech EJ, Coury J, Retecki S (2020). Implementation of a Colorectal Cancer Screening Improvement Program in Community Health Centers: an Applied Use of Configurational Comparative Methods. J Gen Intern Med.

[36] Palinkas LA, Mendon SJ, Hamilton AB. Innovations in mixed methods evaluations. Annu Rev Public Health. 2019;40:423–42. Available from: https://www.annualreviews.org, 10.1146/annurev-publhealth-040218-044215. 10.1146/annurev-publhealth-040218-044215 PMC6501787 30633710

[37] Cragun D. Configurational comparative methods. Handbook on Implementation Science. Cheltenham: Edward Elgar Publishing; 2020. p. 497–504.

[38] Nilsen P, Birken S (2020). Handbook on implementation science.

[39] Ouzzani M, Hammady H, Fedorowicz Z, Elmagarmid A. Rayyan-a web and mobile app for systematic reviews. Syst Rev. 2016;5:1–10. Available from: https://link.springer.com/articles/10.1186/s13643-016-0384-4. 10.1186/s13643-016-0384-4 PMC5139140 27919275

[40] Higgins JPT, Altman DG, Gøtzsche PC, Jüni P, Moher D, Oxman AD, et al. The Cochrane Collaboration’s tool for assessing risk of bias in randomised trials. BMJ. 2011;343. Available from: http://www.bmj.com/content/343/bmj.d5928/suppl/DC1. 10.1136/bmj.d5928 PMC3196245 22008217

[41] Briss PA, Zaza S, Pappaioanou M, Fielding J, Wright-De Agüero L, Truman BI (2000). Developing an evidence-based Guide to Community Preventive Services-Methods. Am J Prev Med.

