# Supplementary material for: Job crafting interventions: what works, for whom, why, and in which contexts? Research protocol for a systematic review with coincidence analysis
Source: Syst Rev. 2023 Jan 20;12:10. doi: 10.1186/s13643-023-02170-z (PMC9857908; doi:10.1186/s13643-023-02170-z)
Supplement: Supplementary file 2 — Additional file 2. Documentation for search strategies. [file 13643_2023_2170_MOESM2_ESM.docx]

Documentation of search strategies

University Library search consultation group

Date: June 2021

Topic/research question: What context, mechanism, and intervention outcomes are relates to successful job crafting interventions?

Name of researcher(s): Marta Rocinewska

Librarian(s): Emma-Lotta Säätelä

Databases:

1. Medline(Ovid)
2. Web of Science Core Collection (Clarivate)
3. Psycinfo (Ovid)
4. Cinahl (EBSCO)

Total number of hits:

- Before deduplication: 4,165
- After deduplication: 2,690

Comments:

PRISMA 2020 flow diagram for new systematic reviews which included searches of databases and registers only^[[1]](#footnote-1)^

**Identification of studies via databases and registers**

Records removed *before screening*:

Duplicate records removed (n = 1,475)

Records marked as ineligible by automation tools (n = )

Records removed for other reasons (n = )

Records identified from*:

Databases

(Medline n = 980

WoS n = 1,688

Psycinfo n = 947

Cinahl n = 570)

Registers (n = )

**Identification**

Records screened

(n = )

Records excluded**

(n = )

Reports sought for retrieval

(n = )

Reports not retrieved

(n = )

**Screening**

Reports assessed for eligibility

(n = )

Reports excluded:

Reason 1 (n = )

Reason 2 (n = )

Reason 3 (n = )

etc.

Studies included in review

(n = )

Reports of included studies

(n = )

**Included**

*Consider, if feasible to do so, reporting the number of records identified from each database or register searched (rather than the total number across all databases/registers).

**If automation tools were used, indicate how many records were excluded by a human and how many were excluded by automation tools.

*From:*  Page MJ, McKenzie JE, Bossuyt PM, Boutron I, Hoffmann TC, Mulrow CD, et al. The PRISMA 2020 statement: an updated guideline for reporting systematic reviews. BMJ 2021;372:n71. doi: 10.1136/bmj.n71

For more information, visit: <http://www.prisma-statement.org/>

1. Medline

| Interface: Ovid MEDLINE(R) and Epub Ahead of Print, In-Process & Other Non-Indexed Citations and Daily  Date of Search: 30 June 2021  Number of hits: 980  Comment: In Ovid, two or more words are automatically searched as phrases; i.e. no quotation marks are needed | Field labels   - exp/ = exploded MeSH term - / = non exploded MeSH term - .ti,ab,kf. = title, abstract and author keywords - adjx = within x words, regardless of order - * = truncation of word for alternate endings |
| --- | --- |
| Database(s): **Ovid MEDLINE(R) and Epub Ahead of Print, In-Process, In-Data-Review & Other Non-Indexed Citations and Daily**1946 to June 29, 2021 Search Strategy:   \| **#** \| **Searches** \| **Results** \| \| --- \| --- \| --- \| \| 1 \| (job demand* resourc* or jd-r).ti,ab,kf. \| 428 \| \| 2 \| (job adj3 (craft* or redesign* or re-design*)).ti,ab,kf. \| 254 \| \| 3 \| ((Seek* or increas* or decreas* or optimiz* or reduc*) adj3 (resource* or demand* or challenge* or hinder*)).ti,ab,kf. \| 57951 \| \| 4 \| (job or workplace* or work-place*).ti,ab,kf. \| 98804 \| \| 5 \| exp Work/ \| 66261 \| \| 6 \| exp Employment/ \| 90995 \| \| 7 \| Professional autonomy/ \| 9599 \| \| 8 \| 4 or 5 or 6 or 7 \| 226267 \| \| 9 \| 3 and 8 \| 1946 \| \| 10 \| 1 or 2 or 9 \| 2514 \| \| 11 \| (intervention* or treatment* or trial* or program* or strateg* or training* or workshop* or exercise* or booklet*).ti,ab,kf. \| 7790363 \| \| 12 \| Program evaluation/ \| 65129 \| \| 13 \| Pamphlets/ \| 3996 \| \| 14 \| 11 or 12 or 13 \| 7806303 \| \| 15 \| 10 and 14 \| 1143 \| \| 16 \| limit 15 to yr="2001 -Current" \| 987 \| \| 17 \| limit 16 to (editorial or letter) \| 7 \| \| 18 \| 16 not 17 \| 980 \| | |

2. Web of Science Core Collection

| Interface: Clarivate Analytics  Date of Search: 30 June 2021  Number of hits: 1,668 | Field labels   - TS/Topic = title, abstract, author keywords and Keywords Plus - NEAR/x = within x words, regardless of order - * = truncation of word for alternate endings   Note: sometimes “quotation marks” are needed for single search terms to avoid automatic term mapping (lemmatization). |
| --- | --- |
| \| **Set** \| **Results** \| **Search** \| \| --- \| --- \| --- \| \| # 7 \| [1,668](https://apps.webofknowledge.com/summary.do?product=WOS&doc=1&qid=138&SID=E5gI1On3vi9FGo8Sjak&search_mode=CombineSearches&update_back2search_link_param=yes) \| #5 AND #4  Refined by: [excluding] DOCUMENT TYPES: ( EDITORIAL MATERIAL OR MEETING ABSTRACT OR BOOK CHAPTER )  *Indexes=SCI-EXPANDED, SSCI, A&HCI, CPCI-S, CPCI-SSH, ESCI Timespan=2001-2021* \| \| # 6 \| [1,793](https://apps.webofknowledge.com/summary.do?product=WOS&doc=1&qid=111&SID=E5gI1On3vi9FGo8Sjak&search_mode=CombineSearches&update_back2search_link_param=yes) \| #5 AND #4  *Indexes=SCI-EXPANDED, SSCI, A&HCI, CPCI-S, CPCI-SSH, ESCI Timespan=All years* \| \| # 5 \| [11,436,643](https://apps.webofknowledge.com/summary.do?product=WOS&doc=1&qid=78&SID=E5gI1On3vi9FGo8Sjak&search_mode=AdvancedSearch&update_back2search_link_param=yes) \| TS=(intervention* or treatment* or trial* or program* or strateg* or training* or workshop* or exercise* or booklet*)  *Indexes=SCI-EXPANDED, SSCI, A&HCI, CPCI-S, CPCI-SSH, ESCI Timespan=All years* \| \| # 4 \| [4,806](https://apps.webofknowledge.com/summary.do?product=WOS&doc=1&qid=110&SID=E5gI1On3vi9FGo8Sjak&search_mode=CombineSearches&update_back2search_link_param=yes) \| #3 OR #2 OR #1  *Indexes=SCI-EXPANDED, SSCI, A&HCI, CPCI-S, CPCI-SSH, ESCI Timespan=All years* \| \| # 3 \| [2,989](https://apps.webofknowledge.com/summary.do?product=WOS&doc=1&qid=109&SID=E5gI1On3vi9FGo8Sjak&search_mode=AdvancedSearch&update_back2search_link_param=yes) \| TS=(((Seek* or increas* or decreas* or optimiz* or reduc*) NEAR/2 (resource* or demand* or challenge* or hinder*) )) AND TS=(("job" or workplace* or "work-place*"))  *Indexes=SCI-EXPANDED, SSCI, A&HCI, CPCI-S, CPCI-SSH, ESCI Timespan=All years* \| \| # 2 \| [812](https://apps.webofknowledge.com/summary.do?product=WOS&doc=1&qid=101&SID=E5gI1On3vi9FGo8Sjak&search_mode=AdvancedSearch&update_back2search_link_param=yes) \| TS=((“Job”) NEAR/2 (craft* or redesign* or "re-design*") )  *Indexes=SCI-EXPANDED, SSCI, A&HCI, CPCI-S, CPCI-SSH, ESCI Timespan=All years* \| \| # 1 \| [1,323](https://apps.webofknowledge.com/summary.do?product=WOS&doc=1&qid=74&SID=E5gI1On3vi9FGo8Sjak&search_mode=AdvancedSearch&update_back2search_link_param=yes) \| TS=(“job demand* resourc*” or “jd-r”)  *Indexes=SCI-EXPANDED, SSCI, A&HCI, CPCI-S, CPCI-SSH, ESCI Timespan=All years* \| | |

3. Psycinfo

| Interface: Ovid  Date of Search: 30 June 2021  Number of hits: 947  Comment: In Ovid, two or more words are automatically searched as phrases; i.e. no quotation marks are needed | Field labels   - exp/ = exploded controlled term - / = non exploded controlled term - .ti,ab,id. = title, abstract and author keywords - adjx = within x words, regardless of order - * = truncation of word for alternate endings |
| --- | --- |
| Database(s): **APA PsycInfo**1806 to June Week 3 2021 Search Strategy:   \| **#** \| **Searches** \| **Results** \| \| --- \| --- \| --- \| \| 1 \| (job demand* resourc* or jd-r).ti,ab,id. \| 962 \| \| 2 \| (job adj3 (craft* or redesign* or re-design*)).ti,ab,id. \| 604 \| \| 3 \| ((Seek* or increas* or decreas* or optimiz* or reduc*) adj3 (resource* or demand* or challenge* or hinder*)).ti,ab,id. \| 16460 \| \| 4 \| (job or workplace* or work-place*).ti,ab,id. \| 120045 \| \| 5 \| exp occupations/ \| 60148 \| \| 6 \| 4 or 5 \| 160782 \| \| 7 \| 3 and 6 \| 1504 \| \| 8 \| 1 or 2 or 7 \| 2824 \| \| 9 \| (intervention* or treatment* or trial* or program* or strateg* or training* or workshop* or exercise* or booklet*).ti,ab,id. \| 1726455 \| \| 10 \| exp intervention/ \| 116042 \| \| 11 \| exp Program Evaluation/ \| 21099 \| \| 12 \| 9 or 10 or 11 \| 1733463 \| \| 13 \| 8 and 12 \| 1078 \| \| 14 \| limit 13 to yr="2001 -Current" \| 962 \| \| 15 \| limit 14 to (editorial or letter or review-book) \| 15 \| \| 16 \| 14 not 15 \| 947 \| | |

4. Cinahl

| Interface: Ebsco  Date of Search: 30 June 2021  Number of hits: 570 | Field labels   - MH+ = exploded Cinahl Heading - MH = non exploded Cinahl Heading - TI = title - AB = abstract - Nx = within x words, regardless of order - * = truncation of word for alternate endings |
| --- | --- |
| \| **#** \| **Query** \| **Results** \| \| --- \| --- \| --- \| \| S15 \| S13 NOT S12  Limiters - Published Date: 20010101-20211231 \| 570 \| \| S14 \| S13 NOT S12 \| 612 \| \| S13 \| S8 AND S11 \| 613 \| \| S12 \| S8 AND S11  Limiters - Publication Type: Book Chapter, Editorial, Letter \| 1 \| \| S11 \| S9 OR S10 \| 2,132,710 \| \| S10 \| (MH "Experimental Studies+") OR (MH "Program Evaluation") OR (MH "Seminars and Workshops+") OR (MH "Pamphlets") \| 432,126 \| \| S9 \| TI ( (intervention* or treatment* or trial* or program* or strateg* or training* or workshop* or exercise* or booklet*) ) OR AB ( (intervention* or treatment* or trial* or program* or strateg* or training* or workshop* or exercise* or booklet*) ) \| 1,999,008 \| \| S8 \| S1 OR S2 OR S7 \| 1,340 \| \| S7 \| S3 AND S6 \| 974 \| \| S6 \| S4 OR S5 \| 124,533 \| \| S5 \| (MH "Work+") OR (MH "Employment+") OR (MH "Professional Autonomy") \| 62,579 \| \| S4 \| TI ( (job or workplace* or "work-place*") ) OR AB ( (job or workplace* or "work-place*") ) \| 73,578 \| \| S3 \| TI ( ((Seek* or increas* or decreas* or optimiz* or reduc*) N2 (resource* or demand* or challenge* or hinder*)) ) OR AB ( ((Seek* or increas* or decreas* or optimiz* or reduc*) N2 (resource* or demand* or challenge* or hinder*)) ) \| 16,032 \| \| S2 \| TI ( ((job) N2 (craft* or redesign* or "re-design*") ) ) OR AB ( ((job) N2 (craft* or redesign* or "re-design*") ) ) \| 184 \| \| S1 \| TI ( (“job demand* resourc*” or “jd-r”) ) OR AB ( (“job demand* resourc*” or “jd-r”) ) \| 254 \| | |

1. 1. Different templates are available depending on the type of review (new or updated) and sources used to identify studies. For more information visit: <http://prisma-statement.org/prismastatement/flowdiagram.aspx> [↑](#footnote-ref-1)
